# Author Correction: Unravelling the spatial directionality of urban mobility

**DOI:** 10.1038/s41467-024-49970-y

**Published:** 2024-07-08

**Authors:** Pengjun Zhao, Hao Wang, Qiyang Liu, Xiao-Yong Yan, Jingzhong Li

**Affiliations:** 1 College of Urban and Environmental Sciences, Peking University, Beijing, 100871 China; 2 School of Urban Planning and Design, Shenzhen Graduate School, Peking University, Shenzhen, 518055 China; 3 School of Systems Science, Beijing Jiaotong University, Beijing, 100044 China; 4 College of Urban and Environmental Sciences, Xuchang University, Xuchang, 461000 China

**Keywords:** Geography, Society

Correction to: *Nature Communications* 10.1038/s41467-024-48909-7, published online 27 May 2024

The original version of this Article contained an error in the Introduction, which incorrectly read:

“We introduce the concept of the population mobility vector (PMV)”.

The correct version now reads as follows, with a citation added to reference 21:

“We introduce the concept of the population mobility vector (PMV)^21^”.

This has been corrected in the PDF and HTML versions of the Article.

