# Supplementary material for: Unravelling the spatial directionality of urban mobility
Source: Nat Commun. 2024 May 27;15:4507. doi: 10.1038/s41467-024-48909-7 (PMC11130278; doi:10.1038/s41467-024-48909-7)
Supplement: Supplementary file 1 — Supplementary Information [file 41467_2024_48909_MOESM1_ESM.pdf]

Supplementary Information
Unravelling the spatial directionality of urban mobility

Pengjun Zhao[1,2,*], Hao Wang[2], Qiyang Liu[2,*], Xiao-Yong Yan[3,*], and Jingzhong Li[4]

[1]*College of Urban and Environmental Sciences, Peking University, Beijing 100871, China*

[2]*School of Urban Planning and Design, Shenzhen Graduate School, Peking University, Shenzhen 518055, China*

[3]*School of Systems Science, Beijing Jiaotong University, Beijing 100044, China*

[4]*College of Urban and Environmental Sciences, Xuchang University, Xuchang 461000, China*

*Corresponding authors: pengjun.zhao@pku.edu.cn, tsql@pku.edu.cn, yanxy@bjtu.edu.cn

Supplementary Figures

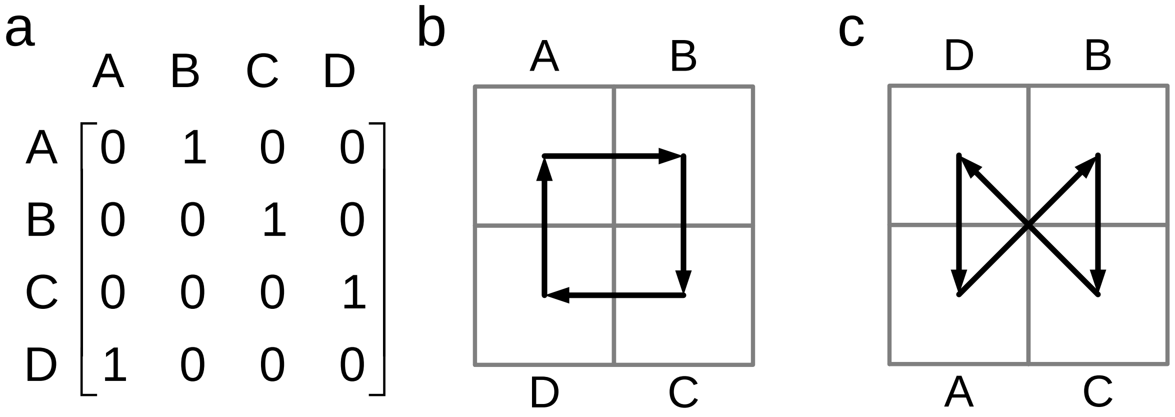

Supplementary Figure 1: **Spatial directionality of urban mobility.** (a) an origin-destination (OD) matrix between four locations: A, B, C and D. (b, c) Two different spatial distributions of the same OD matrix. Two schematic mobility distributions share the same topological structure (i.e., OD matrix), but different spatial layouts result in varying overall characteristics.

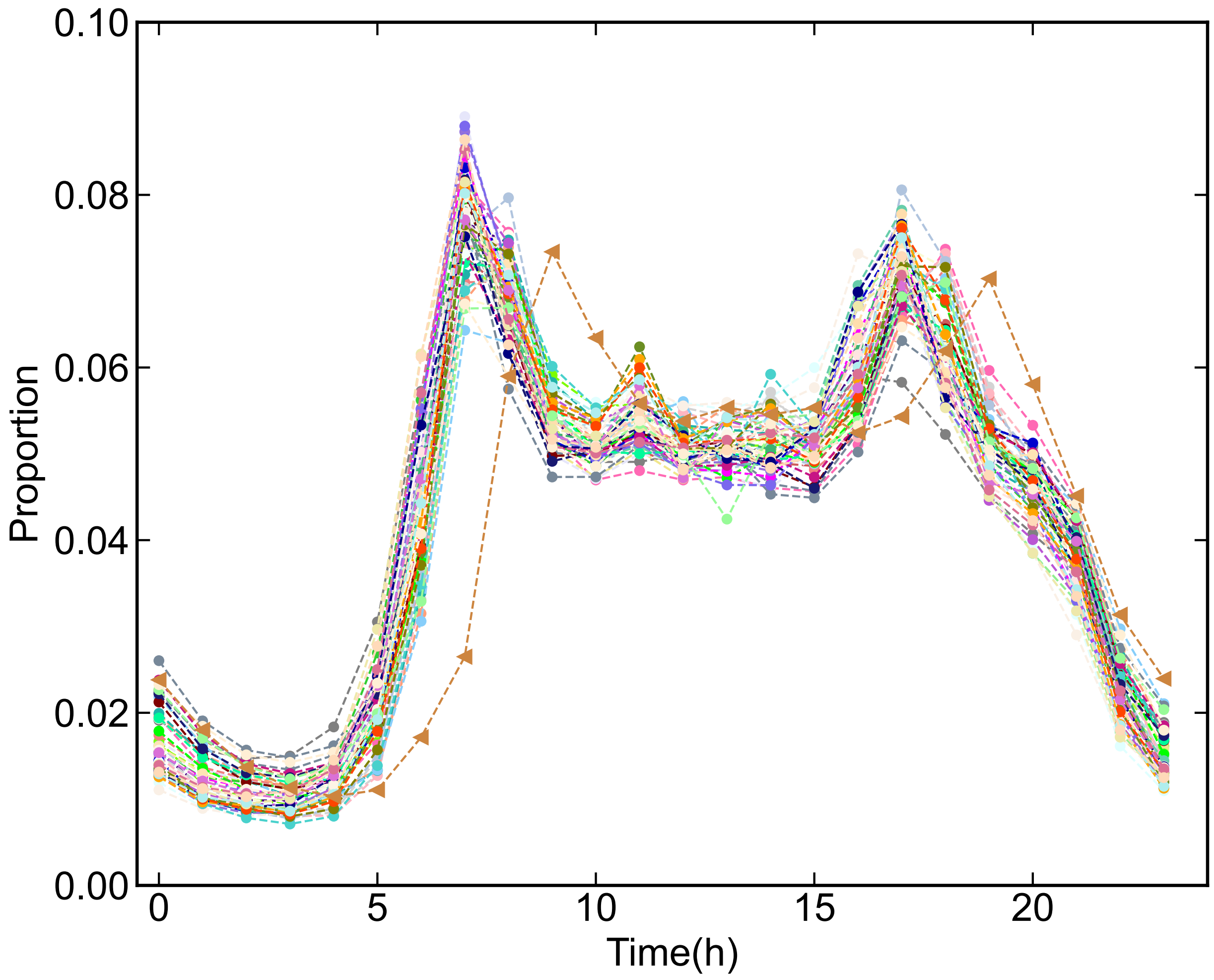

Supplementary Figure 2: **The hourly distribution of flows as a proportion of total daily flows during an average weekday.** Each colour represents data from a distinct city, with each data point indicating the percentage of total hourly flows relative to the city's entire daily flow volume. The brown triangles denote data for the city of Urumqi. Source data are provided as a Source Data file.

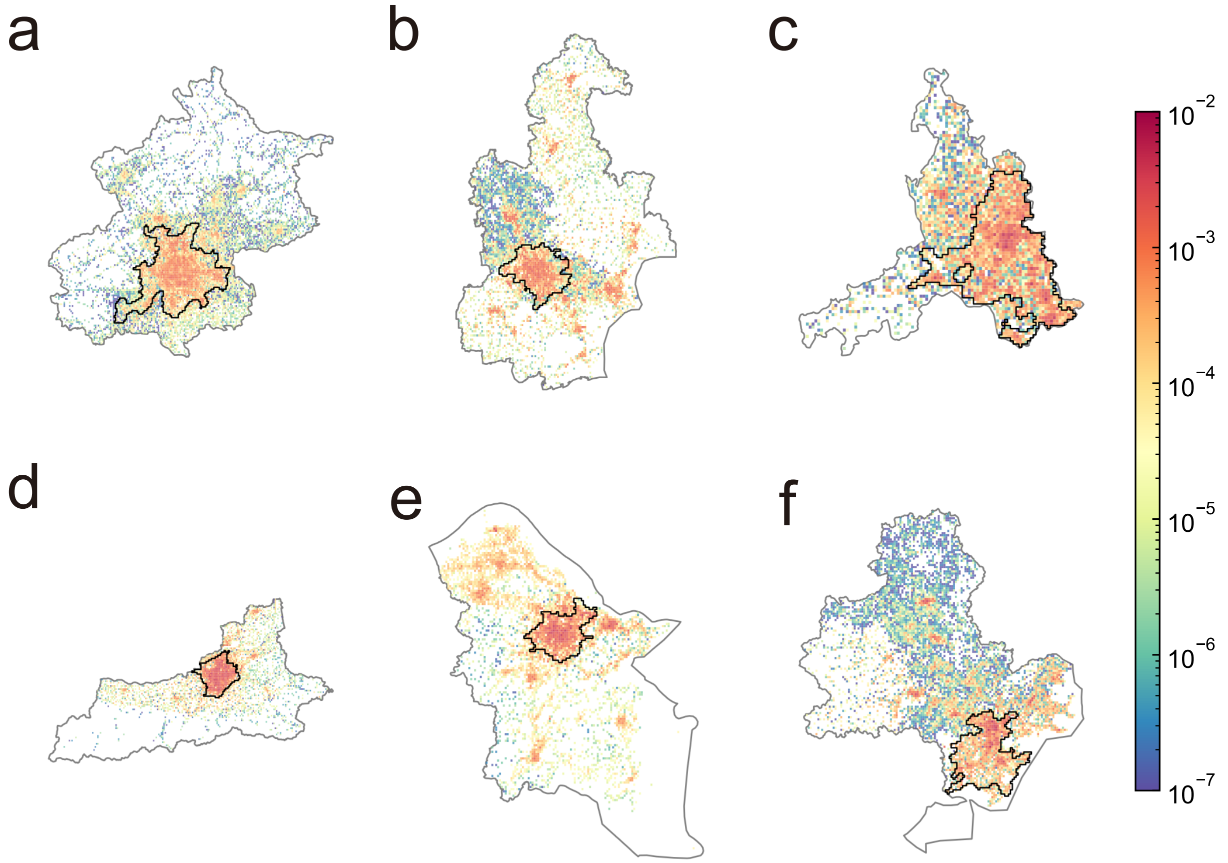

Supplementary Figure 3: **Comparison of the urban area and the outflow density distribution.** (a) Beijing, (b) Tianjin, (c) Foshan, (d) Xian, (e) Ningbo, (f) Quanzhou. The grey line is the administrative boundary and the black line is the boundary of the urban area extracted from the global human settlement dataset. Cells are colored according to their relative outflow (i.e., outflow adjusted by total outflow of all grids within the administrative boundary).

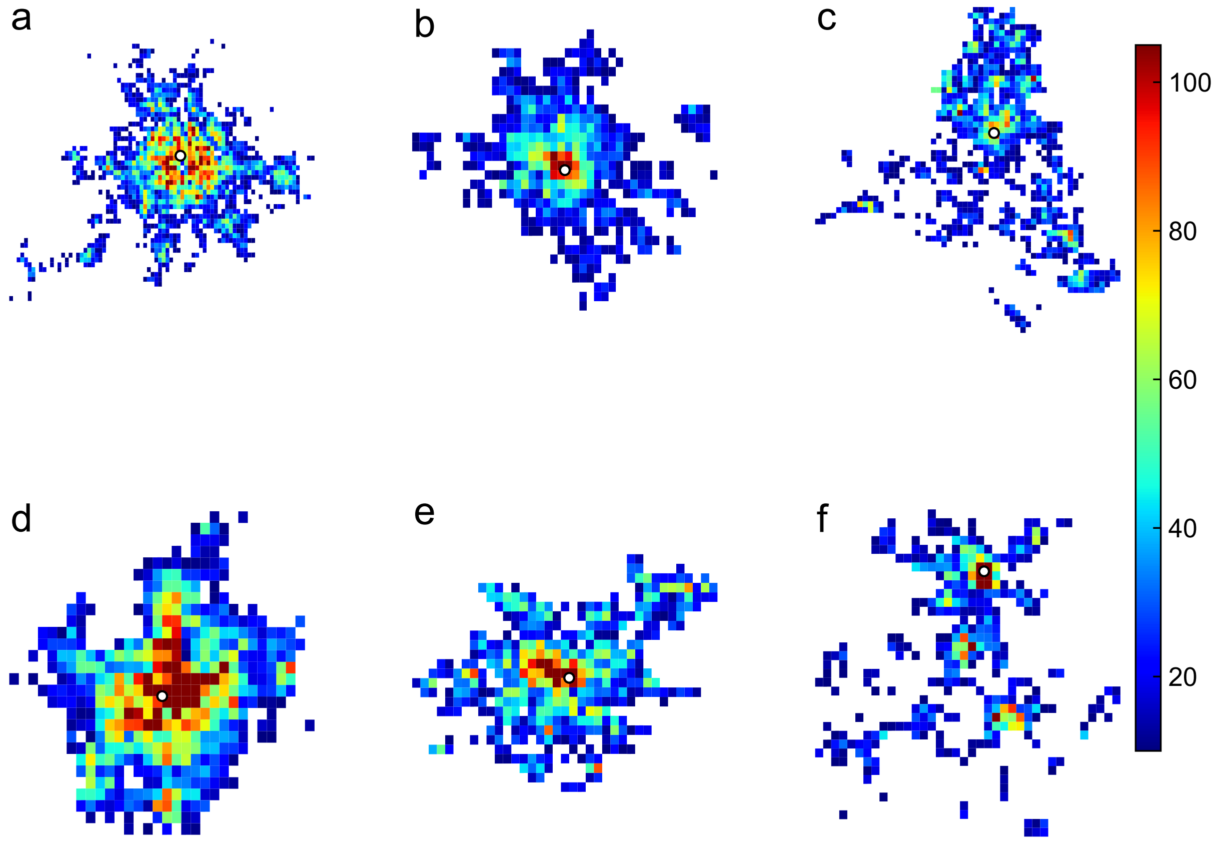

Supplementary Figure 4: **Comparative analysis of the city centre against the distribution of Areas of Interest (AOIs).** The cities featured are (a) Beijing, (b) Tianjin, (c) Foshan, (d) Xi'an, (e) Ningbo and (f) Quanzhou. In each map, the white dot signifies the location of the city centre, with cells shaded according to the number of distinct AOIs they encompass.

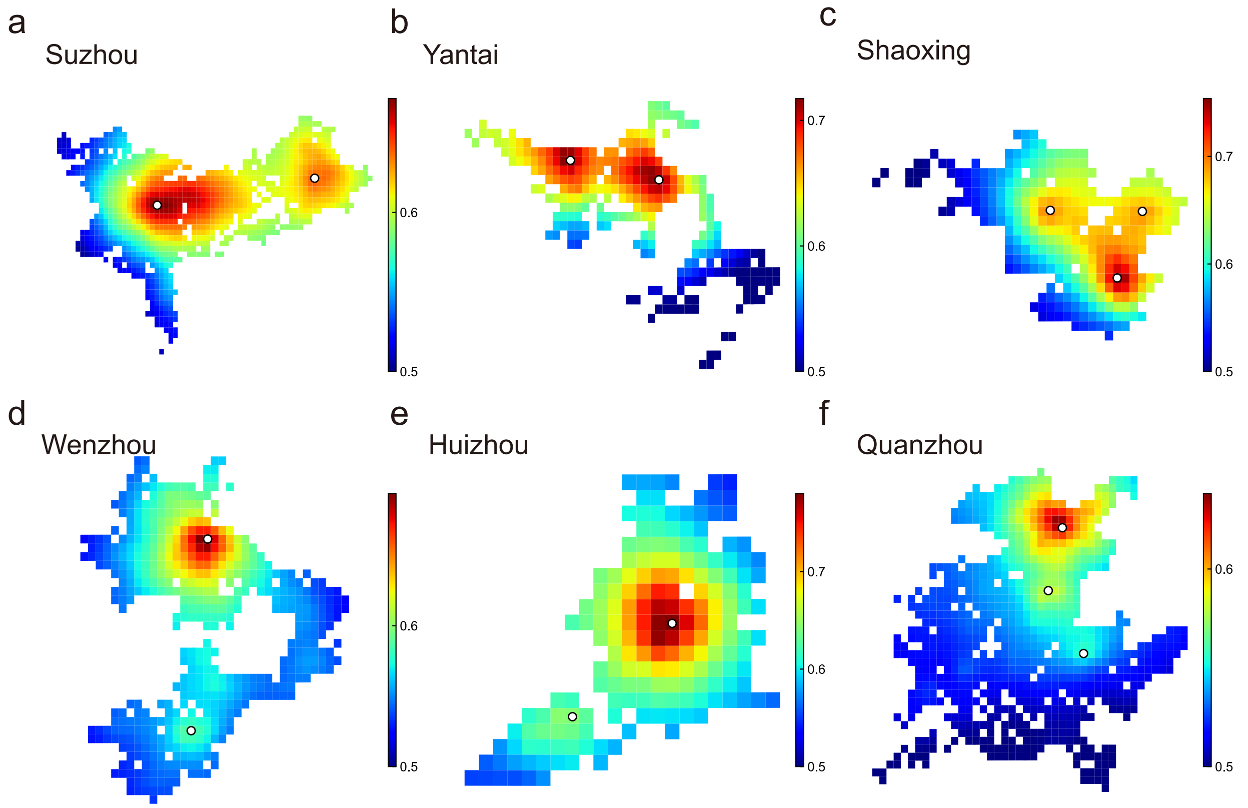

Supplementary Figure 5: **Multiple potential city centre in cities exhibiting polycentric mobility patterns.** The cities analysed include (a) Suzhou, (b) Yantai, (c) Shaoxing, (d) Wenzhou, (e) Huizhou and (f) Quanzhou. In these visualisations, each cell's colour corresponds to the centripetality (Γ) of urban mobility, assuming the cell in question serves as the city centre. The white dots pinpoint the locations of these city centres.

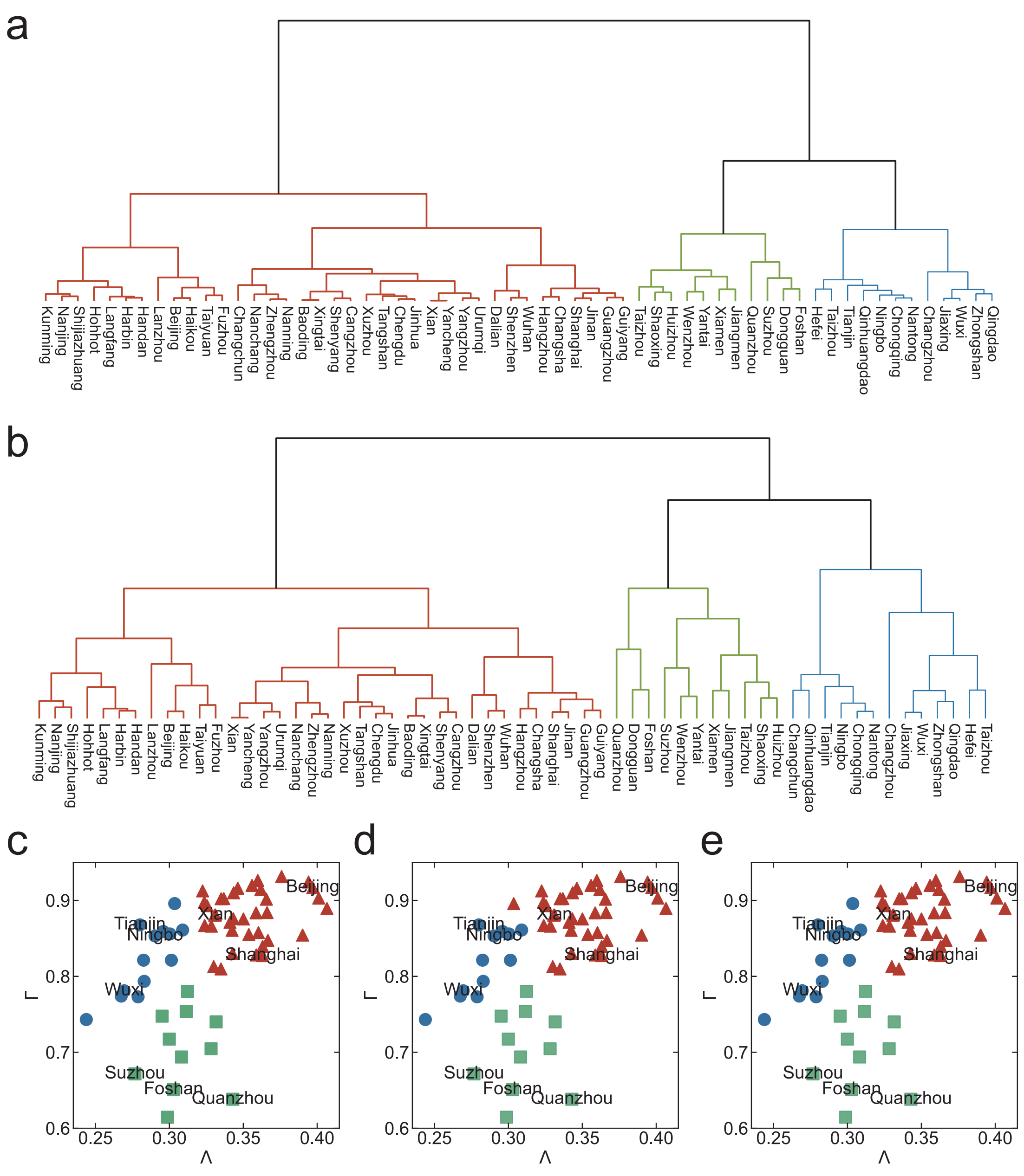

Supplementary Figure 6: **The classification of cities based on different hierarchical clustering methodologies.** Dendrograms generated from the (a) Ward and (b) complete linkage approaches are presented, grounded in the anisotropy and centripetality metrics of commuting flows. Panels (c-e) depict a three-cluster classification of cities, where cities are categorised into three types based on their anisotropy and centripetality values, employing (c) average, (d) Ward and (e) complete linkage approaches respectively. The colour coding within these visualisations signifies the division into three distinct clusters.

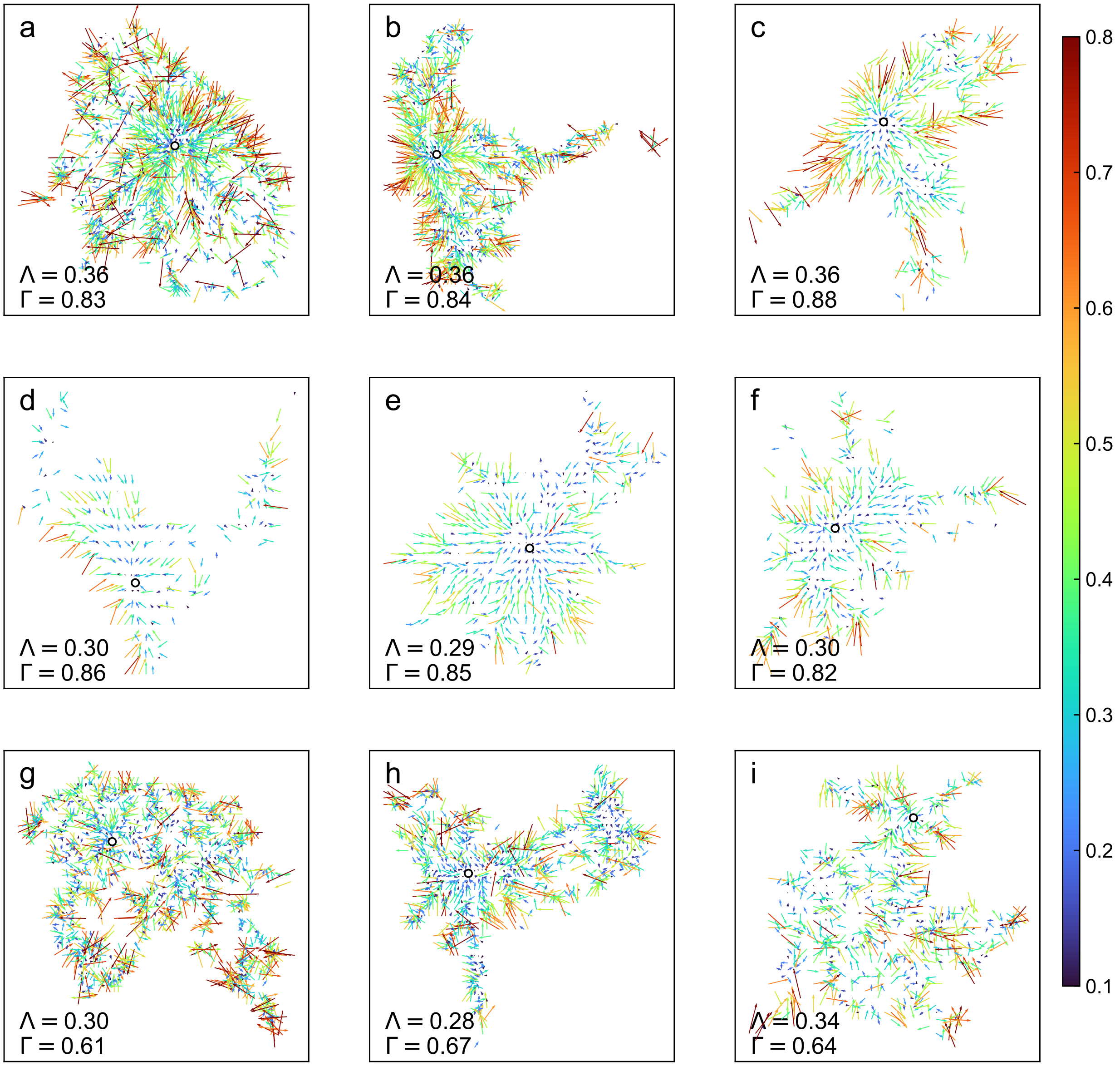

Supplementary Figure 7: **Representative examples of cities referenced in Figure 2 of the main text, showcasing the spatial distribution of Population Mobility Vectors (PMVs).** For strong monocentric cities (a-c), we have (a) Shanghai, (b) Guangzhou and (c) Nanjing; for weak monocentric cities (d-f), the examples are (d) Nantong, (e) Ningbo and (f) Hefei; and for polycentric cities (g-i), the illustrations are (g) Dongguan, (h) Suzhou and (i) Quanzhou. The arrows' length and colour correspond to the PMV magnitude.

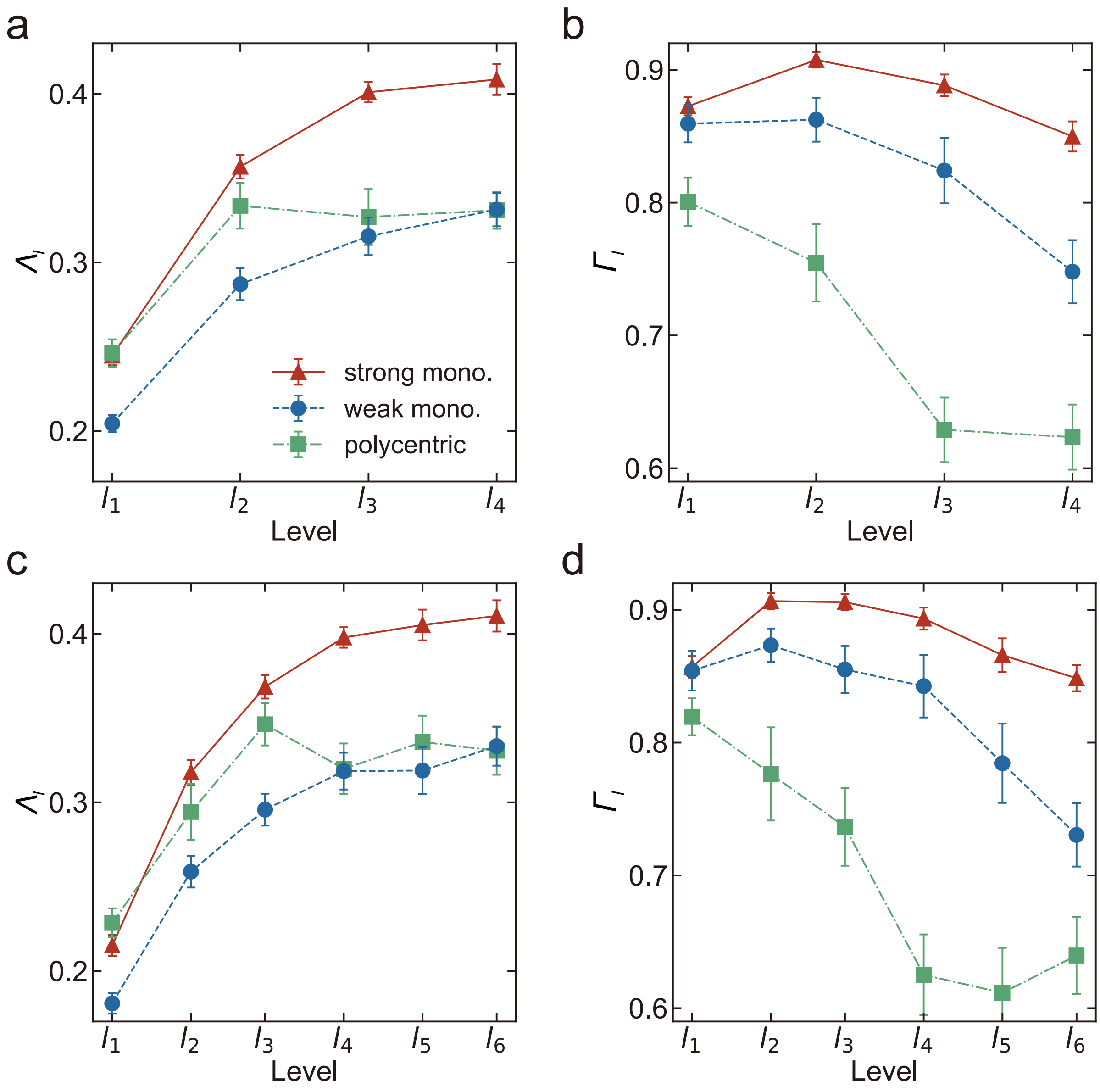

Supplementary Figure 8: **Robustness of the spatial distribution patterns of the anisotropy and centripetality against variations in the level l.** (a-b) The (a) anisotropy and (b) centripetality of four spatial levels for strong monocentric ($n = 36$), weak monocentric ($n = 13$) and polycentric cities ($n = 11$). (c-d) Same as in (a-b) but for six spatial levels. Error bars correspond to standard errors and centre values correspond to the means. Symbols and lines refer to various city types. Source data are provided as a Source Data file.

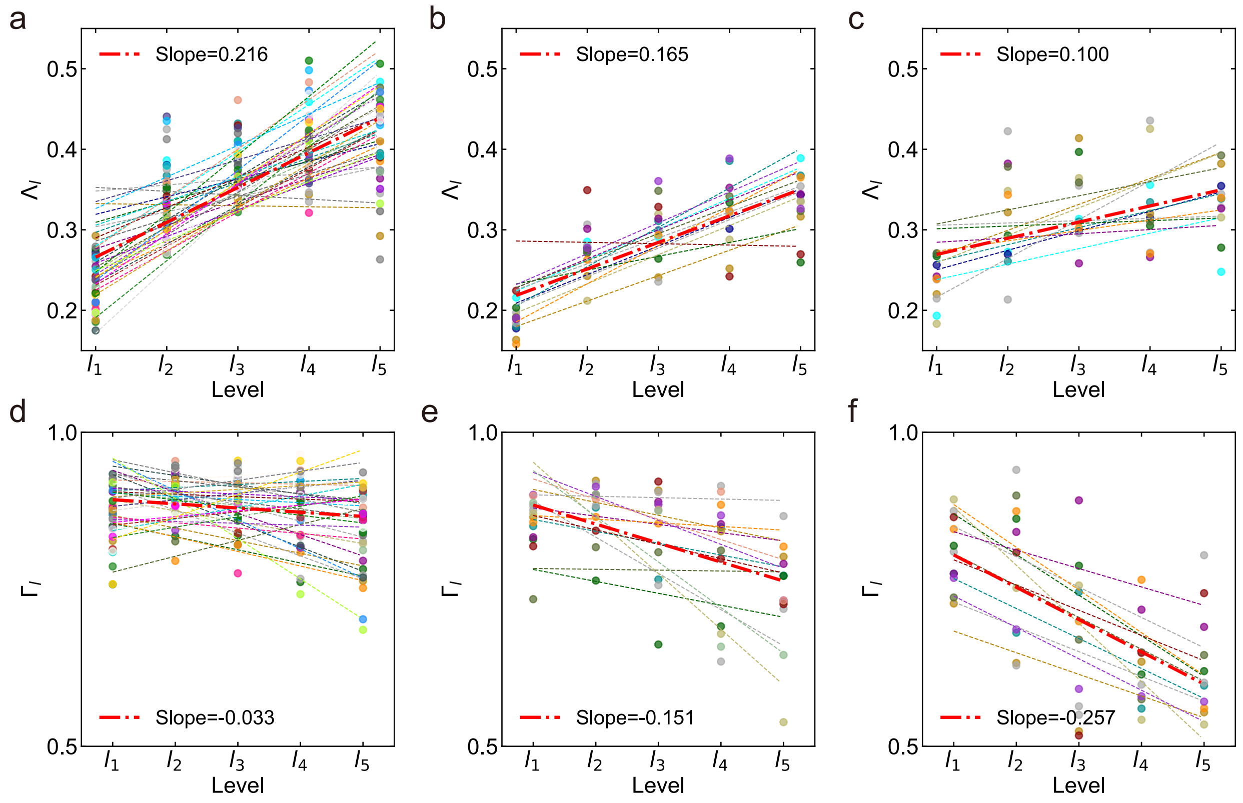

Supplementary Figure 9: **Trends of anisotropy and centripetality within cities.** Panels (a-c) depict the variation in anisotropy across different urban tiers for (a) strong monocentric cities, (b) weak monocentric cities and (c) polycentric cities, with markers representing anisotropy (Λ_l) for each level (distance to the city centre). Each colour symbolises a city, with dashed lines indicating regression lines for each. The red dashed line represents the aggregate linear fit for all data points in each city type. Panels (d-f) convey similar information for centripetality (Γ_l), paralleling the structure of panels (a-c). Source data are provided as a Source Data file.

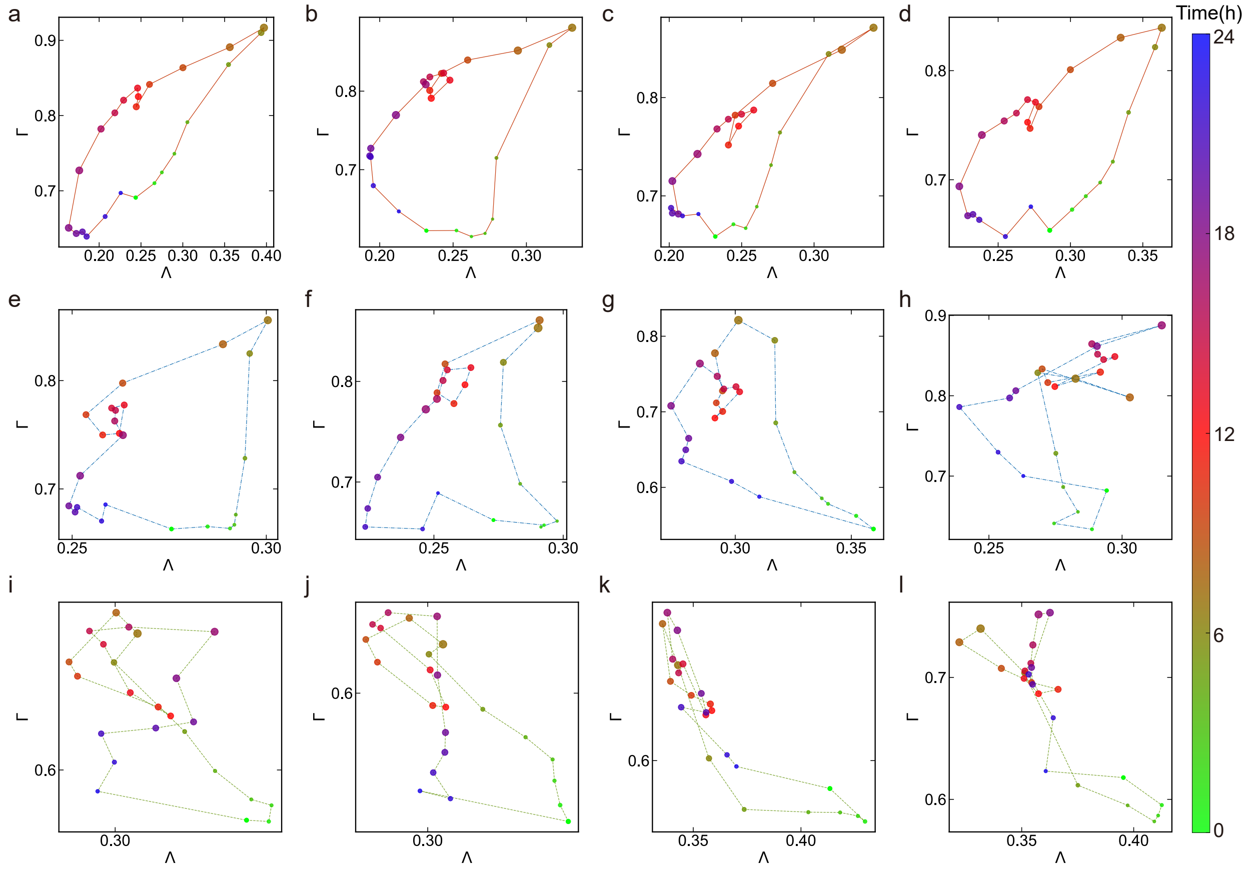

Supplementary Figure 10: **Temporal dynamics of anisotropy and centripetality in urban mobility.** This figure presents the hourly variation in anisotropy and centripetality across cities categorised by urban mobility pattern: (a-d) strong monocentric (Beijing, Xi'an, Chengdu, Guangzhou), (e-h) weak monocentric (Chongqing, Ningbo, Hefei, Taizhou), and (i-l) polycentric (Foshan, Dongguan, Quanzhou, Xiamen). Each point's colour corresponds to a different hour of the day, where green and blue signify early morning and late night, with yellow and red indicating midday. Furthermore, the size of each point reflects the percentage of total hourly flows relative to the city's entire daily travel volume. The lines of different colours and types distinguish between urban mobility patterns. Source data are provided as a Source Data file.

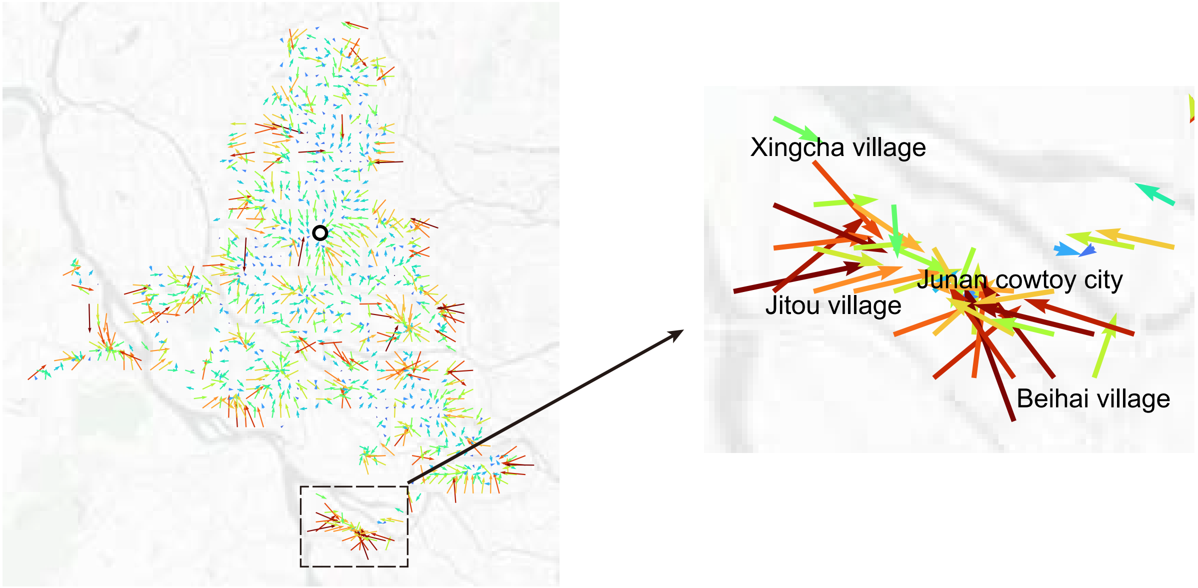

Supplementary Figure 11: **Illustration of the Population Mobility Vectors (PMVs) in Junan.** The left panel displays the PMVs for Foshan and the right panel displays the PMVs for Junan, a town of Foshan. It is evident that the PMVs for Junan are more oriented toward Junan cowtoy city.

Supplementary Tables

Supplementary Table 1: **A comparison between urban area and city area (unit of measurement: square kilometres).**

City	Urban area	City area	Proportion	City	Urban area	City area	Proportion
Beijing	2218	16406	0.135	Changzhou	544	4372	0.124
Shanghai	3004	6341	0.474	Zhongshan	209	1784	0.117
Nanjing	600	6587	0.091	Shaoxing	354	8279	0.043
Shenzhen	1130	1997	0.566	Jiaxing	196	4223	0.046
Guangzhou	1158	7434	0.156	Xuzhou	344	11765	0.029
Hangzhou	1142	16853	0.068	Ningbo	538	9816	0.055
Chengdu	1225	14335	0.085	Wenzhou	612	12110	0.051
Hefei	541	11445	0.047	Wuxi	633	4627	0.137
Suzhou	1149	8657	0.133	Chongqing	448	82402	0.005
Jinan	532	7998	0.067	Hohhot	280	17186	0.016
Wuhan	783	8569	0.091	Huizhou	212	11347	0.019
Changsha	387	11816	0.033	Jiangmen	204	9507	0.021
Tianjin	735	11760	0.063	Yangzhou	151	6591	0.023
Zhengzhou	596	7446	0.08	Nantong	302	10549	0.029
Harbin	518	53076	0.01	Taizhou	118	5787	0.02
Shijiazhuang	598	15848	0.038	Jinhua	123	10942	0.011
Taiyuan	385	6988	0.055	Taizhou	287	10050	0.029
Shenyang	595	12860	0.046	Baoding	249	22185	0.011
Lanzhou	248	13192	0.019	Handan	173	12065	0.014
Xian	563	10958	0.051	Xingtai	130	12433	0.01
Kunming	325	21013	0.015	Cangzhou	158	14304	0.011
Guiyang	278	8043	0.035	Langfang	116	6419	0.018
Nanning	200	22244	0.009	Yancheng	171	16931	0.01
Dongguan	1504	2460	0.611	Dalian	586	13244	0.044
Foshan	1261	3798	0.332	Haikou	179	2289	0.078
Nanchang	389	7402	0.053	Qinhuangdao	122	7803	0.016
Xiamen	426	1701	0.25	Qingdao	599	11282	0.053
Fuzhou	306	12255	0.025	Quanzhou	961	11015	0.087
Tangshan	382	14198	0.027	Yantai	392	13865	0.028
Changchun	450	20594	0.022	Urumqi	363	13788	0.026

Supplementary Table 2: **A comparison between urban population and city population.**

City	Urban population	City population	Proportion	City	Urban population	City population	Proportion
Beijing	17158479	23579690	0.728	Changzhou	2629272	5558454	0.473
Shanghai	27343905	31203967	0.876	Zhongshan	574342	2023502	0.284
Nanjing	6081413	10619553	0.573	Shaoxing	1503759	5549866	0.271
Shenzhen	11035114	11974654	0.922	Jiaxing	924229	5664208	0.163
Guangzhou	12076377	16589349	0.728	Xuzhou	1472710	8276980	0.178
Hangzhou	5120292	9674324	0.529	Ningbo	2879078	9822449	0.293
Chengdu	7641941	16116591	0.474	Wenzhou	4523330	10443470	0.433
Hefei	2772965	7051448	0.393	Wuxi	3107485	7527004	0.413
Suzhou	5050156	11755483	0.43	Chongqing	3782214	26769040	0.141
Jinan	3600159	9349184	0.385	Hohhot	2019896	3480929	0.58
Wuhan	7538878	11458307	0.658	Huizhou	1262870	6185759	0.204
Changsha	3328836	8006136	0.416	Jiangmen	842824	4335754	0.194
Tianjin	7071822	16189555	0.437	Yangzhou	741400	4268525	0.174
Zhengzhou	4942037	11159456	0.443	Nantong	896640	6828066	0.131
Harbin	4329812	12361017	0.35	Taizhou	387496	4253521	0.091
Shijiazhuang	3268020	11387438	0.287	Jinhua	459265	6104421	0.075
Taiyuan	3876707	5376841	0.721	Taizhou	1243473	6701020	0.186
Shenyang	5798014	9310769	0.623	Baoding	1024848	12152759	0.084
Lanzhou	2534533	4221358	0.6	Handan	657165	9597099	0.068
Xian	5127254	9759244	0.525	Xingtai	762290	7981146	0.096
Kunming	3138201	7233182	0.434	Cangzhou	594928	7838674	0.076
Guiyang	2548039	5122444	0.497	Langfang	462727	5072600	0.091
Nanning	1851293	6836684	0.271	Yancheng	407293	6381319	0.064
Dongguan	4245414	4596235	0.924	Dalian	3532343	7560013	0.467
Foshan	4463599	6078082	0.734	Haikou	1427056	2304427	0.619
Nanchang	3200554	6088851	0.526	Qinhuangdao	715047	3281332	0.218
Xiamen	2863959	3794208	0.755	Qingdao	3577324	9862317	0.363
Fuzhou	2995036	7921633	0.378	Quanzhou	4224598	9376982	0.451
Tangshan	1491459	8408669	0.177	Yantai	1268417	7216245	0.176
Changchun	3446865	9651046	0.357	Urumqi	3515176	4298961	0.818

Supplementary Table 3: **The coordinates of the city center identified with the maximum centripetality algorithm.**

City	Longitude	Latitude	City	Longitude	Latitude
Beijing	116.39	39.94	Changzhou	119.96	31.78
Shanghai	121.47	31.22	Zhongshan	113.39	22.52
Nanjing	118.79	32.04	Shaoxing	120.59	30.01
Shenzhen	114.05	22.55	Jiaxing	120.74	30.75
Guangzhou	113.32	23.13	Xuzhou	117.21	34.26
Hangzhou	120.18	30.26	Ningbo	121.58	29.86
Chengdu	104.06	30.64	Wenzhou	120.68	28.00
Hefei	117.26	31.84	Wuxi	120.33	31.55
Suzhou	120.64	31.31	Chongqing	106.54	29.58
Jinan	117.05	36.66	Hohhot	111.70	40.82
Wuhan	114.29	30.59	Huizhou	114.43	23.09
Changsha	113.00	28.18	Jiangmen	113.09	22.59
Tianjin	117.21	39.11	Yangzhou	119.43	32.39
Zhengzhou	113.70	34.77	Nantong	120.89	32.01
Harbin	126.65	45.75	Taizhou	119.93	32.47
Shijiazhuang	114.52	38.04	Jinhua	119.65	29.09
Taiyuan	112.56	37.84	Taizhou	121.43	28.65
Shenyang	123.43	41.79	Baoding	115.49	38.88
Lanzhou	103.84	36.06	Handan	114.51	36.61
Xian	108.94	34.25	Xingtai	114.50	37.07
Kunming	102.73	25.03	Cangzhou	116.85	38.31
Guiyang	106.71	26.58	Langfang	116.72	39.53
Nanning	108.35	22.81	Yancheng	120.16	33.37
Dongguan	113.77	23.01	Dalian	121.62	38.92
Foshan	113.12	23.02	Haikou	110.33	20.02
Nanchang	115.90	28.67	Qinhuangdao	119.59	39.94
Xiamen	118.12	24.5	Qingdao	120.39	36.10
Fuzhou	119.31	26.08	Quanzhou	118.61	24.90
Tangshan	118.18	39.64	Yantai	121.38	37.53
Changchun	125.33	43.88	Urumqi	87.59	43.82

Supplementary Table 4: **The anisotropy and centripetality of each city.**

City	Λ	Γ	City	Λ	Γ
Beijing	0.397	0.917	Changzhou	0.244	0.743
Shanghai	0.363	0.827	Zhongshan	0.279	0.773
Nanjing	0.359	0.884	Shaoxing	0.311	0.754
Shenzhen	0.33	0.813	Jiaxing	0.267	0.774
Guangzhou	0.363	0.839	Xuzhou	0.35	0.876
Hangzhou	0.36	0.857	Ningbo	0.29	0.853
Chengdu	0.341	0.871	Wenzhou	0.308	0.694
Hefei	0.301	0.821	Wuxi	0.269	0.781
Suzhou	0.277	0.672	Chongqing	0.3	0.856
Jinan	0.359	0.828	Hohhot	0.376	0.931
Wuhan	0.335	0.81	Huizhou	0.312	0.78
Changsha	0.354	0.855	Jiangmen	0.328	0.705
Tianjin	0.28	0.868	Yangzhou	0.328	0.866
Zhengzhou	0.324	0.897	Nantong	0.296	0.859
Harbin	0.356	0.92	Taizhou	0.283	0.821
Shijiazhuang	0.366	0.884	Jinhua	0.343	0.876
Taiyuan	0.401	0.903	Taizhou	0.295	0.748
Shenyang	0.346	0.916	Baoding	0.335	0.902
Lanzhou	0.39	0.854	Handan	0.36	0.926
Xian	0.331	0.881	Xingtai	0.337	0.902
Kunming	0.366	0.902	Cangzhou	0.343	0.91
Guiyang	0.367	0.847	Langfang	0.362	0.914
Nanning	0.326	0.893	Yancheng	0.331	0.88
Dongguan	0.299	0.614	Dalian	0.343	0.83
Foshan	0.303	0.651	Haikou	0.394	0.924
Nanchang	0.322	0.912	Qinhuangdao	0.309	0.861
Xiamen	0.332	0.74	Qingdao	0.283	0.793
Fuzhou	0.407	0.889	Quanzhou	0.343	0.638
Tangshan	0.342	0.861	Yantai	0.3	0.717
Changchun	0304	0.896	Urumqi	0.324	0.867

Supplementary Table 5: **Statistical indicators of daily variations in anisotropy and centripetality.**

Mobility patterns	Range of Γ	SD of Γ	Range of Λ	SD of Λ
strong monocentric	0.218	0.069	0.107	0.028
weak monocentric	0.181	0.063	0.041	0.012
polycentric	0.115	0.039	0.041	0.012

Supplementary Table 6: **Statistical indicators of anisotropy and centripetality changes over 4 to 8 hours.**

Mobility patterns	Change of Γ	Change rate of Γ	Change of Λ	Change rate of Λ
strong monocentric	0.168	23.6%	0.031	10.4%
weak monocentric	0.149	22.0%	0.004	1.3%
polycentric	0.095	15.4%	-0.018	-5.7%

Supplementary Notes

Supplementary Note 1: Illustrating the importance of spatial directionality in mobility

Supplementary Figure 1 presents that a mobility network with the same OD matrix can have multiple distinct layouts, indicating the spatial information has fundamental connections with the overall characteristics of human mobility. To incorporate the spatial directionality associated with human mobility, we first vectorize the flow T_{ij} and obtain a vector $T_{ij}\overrightarrow{u_{ij}}$, where $\overrightarrow{u_{ij}}$ is the unit vector from i to j. Then, to factor out the effects of the variation in outflow across locations, we rescale $T_{ij}\overrightarrow{u_{ij}}$ by the outflow of its origin, giving $\overrightarrow{T_{ij}} = T_{ij}\overrightarrow{u_{ij}}/O_i$. Finally, we sum the vectors pointing to all destinations j and obtain the PMV $\overrightarrow{T_i}$, as shown in Figure 1 in the main text. It is worth noting that the PMV coincides mathematically with the vector field for commuting mobility [1]. This coincidence further suggests the rationality of the PMV definition.

Supplementary Note 2: Characterising the typical morning peak hour

Supplementary Figure 2 depicts the hourly variation in trip volumes as a percentage of each city's total daily trips. Universally, cities display two peak periods: morning peak volumes peak between 7 and 8 a.m., and evening peaks between 5 and 6 p.m.. The convergence of data from various cities suggests a standardised temporal pattern across urban environments, attributed to the regimented work and rest schedules prevalent in contemporary society, fostering rhythmic urban lifestyles and consistent mobility trends. A unique observation is that Urumqi, located in Western China and marked by triangles in the figure, experiences peak periods about two hours later than other cities. Due to the pronounced regularity of morning commuting patterns and their significance in addressing transportation supply-demand conflicts, this study utilises data from the typical morning peak hour (7 to 8 a.m., adjusted to 9 to 10 a.m. for Urumqi) as the foundation for analysing mobility direc-

tionality and for clustering cities. This peak hour's trip volume represents approximately 8% of the total daily trips (Supplementary Figure 2).

Supplementary Note 3: Extracting the urban area

The urban area, an essential spatial property, is widely used in many urban studies such as urban planning and assessment of urban development. However, the definition of urban areas varies with different countries and applications. For example, the conventional method of defining urban areas in the USA is the Metropolitan Statistical Areas, while that in Europe is the Larger Urban Zones [2]. These definitions are inconsistent with one another as no consensus exists on how urban areas should be defined [3]. Note that population is a commonly used indicator for defining human agglomerations. We therefore adopt the global human settlement dataset [4], which defines the urban area as a contiguous area with a density of at least 1,500 inhabitants per square kilometer or a majority of built-up land cover coinciding with a minimum of 50,000 inhabitants, to extract the urban area and corresponding population. Supplementary Figure 3 depicts the boundary of the urban area (black line) together with the density distribution of outflow per grid in six selected cities. As can be seen, most of the cells with a large number of outflow are located within the urban area, indicating that the mobile phone dataset and the urban area are highly coincidental.

Supplementary Table 1 offers an in-depth comparison of urban area sizes, city area sizes as delineated by administrative boundaries, and the urban to city area size ratio for each city. In parallel, Supplementary Table 2 details urban and city population sizes, alongside the urban population's share of the total city population. Notably, the urban to city area ratio exhibits substantial variation across cities, ranging from as little as one percent to over fifty percent, with an average around 8.1%. This variation highlights significant disparities between administrative and actual urban extents, emphasising the need for precise urban area delineation in research. On the other hand, urban populations constitute, on average, 40% of total city populations, indicating that urban zones are primary centres of human concentration. The discrepancy between the compact spatial footprint of urban areas and their dense population emphasises urban regions' role as focal points of human activity, reinforcing the importance of accurately identifying these areas to enhance our understanding of urban mobility patterns and dynamics.

Supplementary Note 4: Identification results of the city center

Supplementary Table 3 displays the city centre as determined by the Maximum Centripetality Algorithm (MCA). This algorithm offers a systematic and automated method for identifying the city centre based on human mobility data, thereby circumventing the need for subjective definitions or reliance on mapping services. The city centre, as defined by our approach, reflects the socioeconomic nucleus of interactions, anchored in physical space through human movements.

The validity of the identified city centre is further corroborated by integrating AOI data, illustrated in Supplementary Figure 4. A notable aggregation of AOIs around the city centre corroborates its accuracy and reliability, suggesting the presence of a discernible pattern of land use activity revealed through mobile phone data. These findings not only affirm the credibility of our method in pinpointing the city centre but also enrich our analysis by highlighting the interplay between human activity patterns and urban land use dynamics.

Our research initially targets the identification of a singular city centre within urban areas, a method proven effective for elucidating urban mobility patterns. However, we acknowledge that polycentric cities possess multiple centres, diverging from the monocentric model. Extending beyond the determination of an optimal city centre, our study also discerns the presence of additional centres within such cities. Supplementary Figure 5 illustrates cities exhibiting polycentric mobility patterns, with each location colour-coded according to its centripetality measure (Γ) when considered as a potential city centre. In these cities, we observe distinct regions with substantial centripetal forces, indicating the formation of multiple urban core areas. This suggests that alongside the primary city centre, there are secondary centres of urban activity. We identify these suboptimal city centres, or subcentres, located in proximity to the main city core, through qualitative analysis. This analysis unequivocally indicates multiple city centres, challenging the traditional notion of a singular focal point. Recognising and analysing the dispersion of these centres offers critical insights for urban planning, mobility management, and fostering sustainable urban spaces.

Supplementary Note 5: Classification of cities

We first calculate the anisotropy and centripetality of each city during the typical morning peak hour, as detailed in Supplementary Table 4. Subsequently, we apply the hierarchical clustering method to classify cities based on their similarity (distance) in terms of their anisotropy and centripetality. There are different approaches of hierarchical clustering, such as average linkage, Ward linkage and complete linkage [5]. For the average linkage approach, the distance between two clusters was calculated as the average distances between all pairs of cities in two clusters. For the Ward linkage approach, it was based on a classical sum-of-squares criterion, producing clusters that minimize the increase in the within-cluster sum of squares at each binary fusion. For the complete linkage approach, the distance between clusters was calculated as the maximum distance among each pair of cities in two clusters. In the main text, we used the average linkage approach and found that three clusters give the best interpretation (Figure 3). Additionally, we also used the Ward linkage approach and complete linkage to classify cities and find that the results are almost the same, suggesting this three-clusters classification is robust and appropriate(Supplementary Figure 6).

Supplementary Note 6: Spatial distribution of PMVs in different city clusters

In the main paper, we explore the mobility characteristics and patterns across different city types. Building on this, Supplementary Figure 7 showcases examples for each of the three identified city types, reinforcing the validity of our classification and interpretation. This evidence indicates that cities classified within the same category share comparable PMV patterns, whereas cities from different categories exhibit unique PMV configurations. Specifically, strong monocentric cities are characterised by a high degree of anisotropy and centripetality, as illustrated in Supplementary Figures 7a-c. In contrast, polycentric cities are marked by lower levels of both anisotropy and centripetality (Supplementary Figures 7g-i), whilst weak monocentric cities manifest a mixed pattern of low anisotropy coupled with high centripetality (Supplementary Figures 7d-f). These observations validate the coherence of our quantified metrics with the mobility pattern-based categorisation, underscoring the

robustness and reliability of both our metric quantification and the classification framework.

Supplementary Note 7: Spatial hierarchical structure of cities

To analyse cities within a unified framework, inspired by the work of Bertaud and Malpezzi [6], we defined a series of concentric rings centred at the city centre, aligning with the structural characteristics of many Chinese cities. To accommodate rapid urbanisation, population growth, and increasing transportation demands, numerous cities have developed multi-ring road networks, with the five-ring structure being particularly emblematic. Given the varying sizes of cities, employing a constant distance to segment cities is impractical. Instead, we adopted a criterion based on equal trip generation (which correlates with population due to the proportional relationship between trip generation and population density [7]) to divide cities uniformly into “ring zones”, ensuring each ring encompasses an equal number of generated trips (and by extension, population).

In the main text, we use $l = 5$ rings for all cities, while our results are robust against changes of this value. As shown in Supplementary Figure 8, there is no quantitative change in our results when varying the number of spatial levels l. The anisotropy still increases with the spatial level across city types and the centripetality still decreases with the spatial level across city types. Furthermore, despite these regularities are discovered at the city-average level, they are still detected at the single-city level (Supplementary Figure 9), which confirms that the observed regularities are intrinsic and universal properties of urban mobility. It is worth mentioning that the centripetality of all spatial levels in all these cities is greater than 0.5 (Supplementary Figures 9d-f), indicating that, on average, the dominant mobility consistently gravitates towards the urban center (urban core). This result points to the strong influence of urban core, which has an overall attraction effect on all mobility flows in urban area.

The dashed red lines in Supplementary Figure 9 depict the linear regression results for all points representing city anisotropy or centripetality for each city type. The slopes indicate that, in terms of anisotropy, strong monocentric cities still experience the rapid increase, followed by weak monocentric cities, and finally polycentric cities (Supplementary Figures 9a-c). For centripetality, strong monocentric cities still experience the slowest decrease, followed by

weak monocentric cities, and finally polycentric cities (Supplementary Figures 9d-f). Moreover, it is observed that the centripetality of some strong monocentric cities increases with spatial level (Supplementary Figure 9d), indicating that the urban core of these cities has a strong attraction, increasingly drawing people towards the city centre. These results further confirm the validity of our classification for the three types of cities based on anisotropy and centripetality.

Supplementary Note 8: The anisotropy and centripetality of a spatial level

The anisotropy of a spatial level l ($l = l_1, l_2, l_3, l_4, l_5$) is computed as the weighted average of each location's anisotropy, with the outflow of each location as a weight, i.e.,

$$\Lambda_l = \frac{\sum_{i \in l} O_i \lambda_i}{\sum_{i \in l} O_i}, \tag{1}$$

where Λ_l is the anisotropy of the spatial level l and $i \in l$ denotes i is located in the spatial level l.

In the same way, the centripetality of the spatial level l is computed as the weighted average of each location's centripetality, with the outflow of each location as a weight, i.e.,

$$\Gamma_l = \frac{\sum_{i \in l} O_i \gamma_i}{\sum_{i \in l} O_i}, \tag{2}$$

where Γ_l is the anisotropy of the spatial level l and $i \in l$ denotes i is located in the spatial level l.

Supplementary References

[1] Mazzoli, M. *et al.* Field theory for recurrent mobility. *Nat. Commun.* **10**, 3895 (2019).

[2] Bettencourt, L. M. A. & Lobo, J. Urban scaling in Europe. *J. R. Soc. Interface.* **13**, 20160005 (2016).

[3] Barthelemy, M. The statistical physics of cities. *Nat. Rev. Phys.* **1**, 406–415 (2019).

[4] Florczyk, A. *et al.* GHS-UCDB R2019A—GHS Urban Centre Database 2015, multitemporal and multidimensional attributes. European Commission, Joint Research Centre. https://data.jrc.ec.europa.eu/dataset/53473144-b88c-44bc-b4a3-4583ed1f547e (accessed April 13, 2022)

[5] Govender, P. & Sivakumar, V. Application of k-means and hierarchical clustering techniques for analysis of air pollution: A review (1980–2019). *Atmos. Pollut. Res.* **11**, 40-56 (2020).

[6] Bertaud, A. & Malpezzi, S. *The Spatial Distribution of Population in 48 World Cities: Implications for Economies in Transition* (The Center for Urban Land Economics Research, University of Wisconsin, 2003).

[7] Simini, F., González, M. C., Maritan, A. & Barabási, A. L. A universal model for mobility and migration patterns. *Nature* **484**, 96-100 (2012).
