# Supplementary material for: Unravelling the spatial directionality of urban mobility
Source: Nat Commun. 2024 May 27;15:4507. doi: 10.1038/s41467-024-48909-7 (PMC11130278; doi:10.1038/s41467-024-48909-7)
Supplement: Supplementary file 3 — Reporting Summary [file 41467_2024_48909_MOESM3_ESM.pdf]

nature portfolio

Corresponding author(s): Pengjun Zhao, Qiyang Liu, Xiao-Yong Yan

Last updated by author(s): Apr 21, 2024

Reporting Summary

Nature Portfolio wishes to improve the reproducibility of the work that we publish. This form provides structure for consistency and transparency in reporting. For further information on Nature Portfolio policies, see our Editorial Policies and the Editorial Policy Checklist.

Statistics

For all statistical analyses, confirm that the following items are present in the figure legend, table legend, main text, or Methods section.

n/a	Confirmed	
☐	☒	The exact sample size (n) for each experimental group/condition, given as a discrete number and unit of measurement
☐	☒	A statement on whether measurements were taken from distinct samples or whether the same sample was measured repeatedly
☐	☒	The statistical test(s) used AND whether they are one- or two-sided<br>*Only common tests should be described solely by name; describe more complex techniques in the Methods section.*
☒	☐	A description of all covariates tested
☐	☒	A description of any assumptions or corrections, such as tests of normality and adjustment for multiple comparisons
☐	☒	A full description of the statistical parameters including central tendency (e.g. means) or other basic estimates (e.g. regression coefficient) AND variation (e.g. standard deviation) or associated estimates of uncertainty (e.g. confidence intervals)
☐	☒	For null hypothesis testing, the test statistic (e.g. F, t, r) with confidence intervals, effect sizes, degrees of freedom and P value noted<br>*Give P values as exact values whenever suitable.*
☒	☐	For Bayesian analysis, information on the choice of priors and Markov chain Monte Carlo settings
☒	☐	For hierarchical and complex designs, identification of the appropriate level for tests and full reporting of outcomes
☒	☐	Estimates of effect sizes (e.g. Cohen's d, Pearson's r), indicating how they were calculated

Our web collection on statistics for biologists contains articles on many of the points above.

Software and code

Policy information about availability of computer code

Data collection	No data collection was performed for this study; all analysis relied on previously collected datasets, as described in the Data section below.
Data analysis	The analysis was conducted using Python. (V.3.8, Python Software Foundation). Code is available at https://codeocean.com/capsule/9118584/tree/v1

For manuscripts utilizing custom algorithms or software that are central to the research but not yet described in published literature, software must be made available to editors and reviewers. We strongly encourage code deposition in a community repository (e.g. GitHub). See the Nature Portfolio guidelines for submitting code & software for further information.

Data

Policy information about availability of data

All manuscripts must include a data availability statement. This statement should provide the following information, where applicable:

- Accession codes, unique identifiers, or web links for publicly available datasets
- A description of any restrictions on data availability
- For clinical datasets or third party data, please ensure that the statement adheres to our policy

The authors declare that the data supporting the conclusions in the paper are described in the paper and the Supplementary Information. The mobile phone data are not publicly accessible because of privacy and contractual concerns. Information about the conditions and limitations of the data can be found in http://www.smartsteps.com/product/dataset.php. Submit your data access request by contacting the sales team via the customer page at http://www.smartsteps.com/about/contactus.php, where they will promptly facilitate your request process. Source data are provided as a Source Data file.

Research involving human participants, their data, or biological material

Policy information about studies with human participants or human data. See also policy information about sex, gender (identity/presentation), and sexual orientation and race, ethnicity and racism.

Reporting on sex and gender	NA
Reporting on race, ethnicity, or other socially relevant groupings	NA
Population characteristics	We used the aggregated mobile data of the Chinese phone users.
Recruitment	NA Population flow data was provided in aggregate form by a major Chinese carrier.
Ethics oversight	This work has been supported by the National Natural Science Foundation of China. We do not use individual-level data, only anonymized aggregate flows, and this work is exempt from IRB review in China.

Note that full information on the approval of the study protocol must also be provided in the manuscript.

Field-specific reporting

Please select the one below that is the best fit for your research. If you are not sure, read the appropriate sections before making your selection.

☐ Life sciences ☒ Behavioural & social sciences ☐ Ecological, evolutionary & environmental sciences

For a reference copy of the document with all sections, see nature.com/documents/nr-reporting-summary-flat.pdf

Behavioural & social sciences study design

All studies must disclose on these points even when the disclosure is negative.

Study description	This is a quantitative study. We apply vector computation to unravel the spatial directionality of urban mobility. We introduce the concept of the population mobility vector, which naturally leads to the development of two key metrics: anisotropy and centripetality. These metrics adeptly capture the imbalances in direction distribution and the orientation towards the city centre in mobility flows. Employing these metrics, we can precisely quantify the unique directional characteristics of cities, enabling us to classify urban areas and to dissect their distinct mobility patterns.
Research sample	The dataset consists of mobile phone signal records collected over a two-month period, representing approximately 90 million residents across 60 cities. These sample cities predominantly consist of directly administered municipalities and provincial capitals with superior economic development, thereby rendering them representative and capable of illustrating the mobility characteristics of China's major urban centers. The number of anonymised users represents on average 18% of the total population. Sex and age were not collected.
Sampling strategy	We did not perform sampling, sample size was determined by the size of the operator's database.
Data collection	We did not perform data collection, but relied on previously collected, anonymized data by the telecommunications operator.
Timing	The aggregated, nonpersonally identifiable human movement records represent the number of trips between different grids per hour<br>in August and November 2019.
Data exclusions	No data were excluded from the analyses.
Non-participation	No participations dropped out.
Randomization	This study was not an experiment, and it did not have experimental conditions.

Reporting for specific materials, systems and methods

We require information from authors about some types of materials, experimental systems and methods used in many studies. Here, indicate whether each material, system or method listed is relevant to your study. If you are not sure if a list item applies to your research, read the appropriate section before selecting a response.

Materials & experimental systems

n/a	Involved in the study
☒	☐ Antibodies
☒	☐ Eukaryotic cell lines
☒	☐ Palaeontology and archaeology
☒	☐ Animals and other organisms
☒	☐ Clinical data
☒	☐ Dual use research of concern
☒	☐ Plants

Methods

n/a	Involved in the study
☒	☐ ChIP-seq
☒	☐ Flow cytometry
☒	☐ MRI-based neuroimaging

Plants

Seed stocks	*Report on the source of all seed stocks or other plant material used. If applicable, state the seed stock centre and catalogue number. If plant specimens were collected from the field, describe the collection location, date and sampling procedures.*
Novel plant genotypes	*Describe the methods by which all novel plant genotypes were produced. This includes those generated by transgenic approaches, gene editing, chemical/radiation-based mutagenesis and hybridization. For transgenic lines, describe the transformation method, the number of independent lines analyzed and the generation upon which experiments were performed. For gene-edited lines, describe the editor used, the endogenous sequence targeted for editing, the targeting guide RNA sequence (if applicable) and how the editor was applied.*
Authentication	*Describe any authentication procedures for each seed stock used or novel genotype generated. Describe any experiments used to assess the effect of a mutation and, where applicable, how potential secondary effects (e.g. second site T-DNA insertions, mosiacism, off-target gene editing) were examined.*
